# Supplementary figures and images for: Identification of Arhgef12 and Prkci as genetic modifiers of retinal dysplasia in the Crb1rd8 mouse model
Source: PLoS Genet. 2022 Jun 8;18(6):e1009798. doi: 10.1371/journal.pgen.1009798 (PMC9212170; doi:10.1371/journal.pgen.1009798)

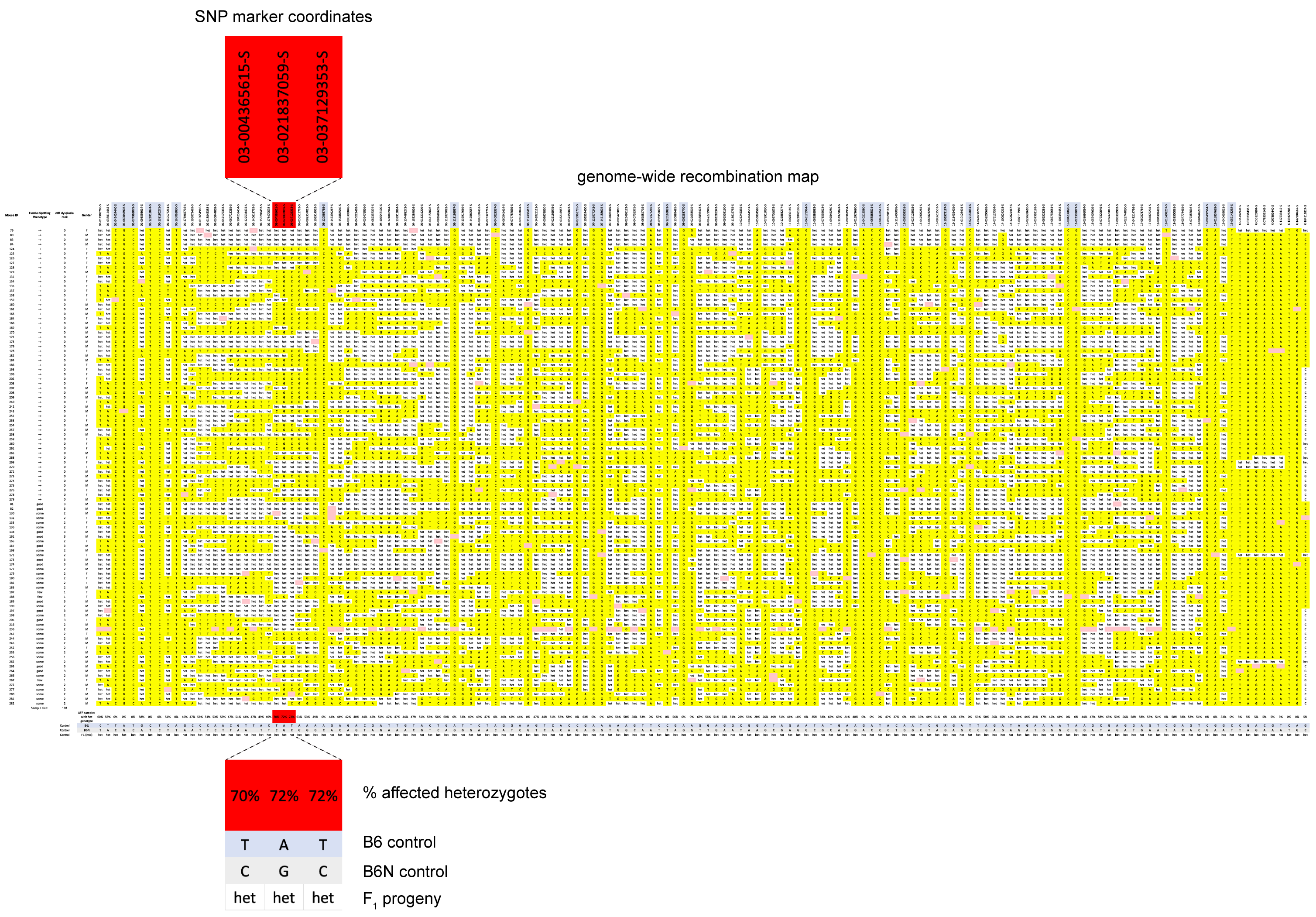

Supplement: S1 Fig — Each column indicates a SNP and is ordered based on chromosome position. Homozygosity of the B6N allele is shown in yellow. The critical interval of the disease gene lies in the region highlighted in red, where ≥70% of the affected progeny were heterozygous for the corresponding SNP. (TIF) [file pgen.1009798.s001.tif]

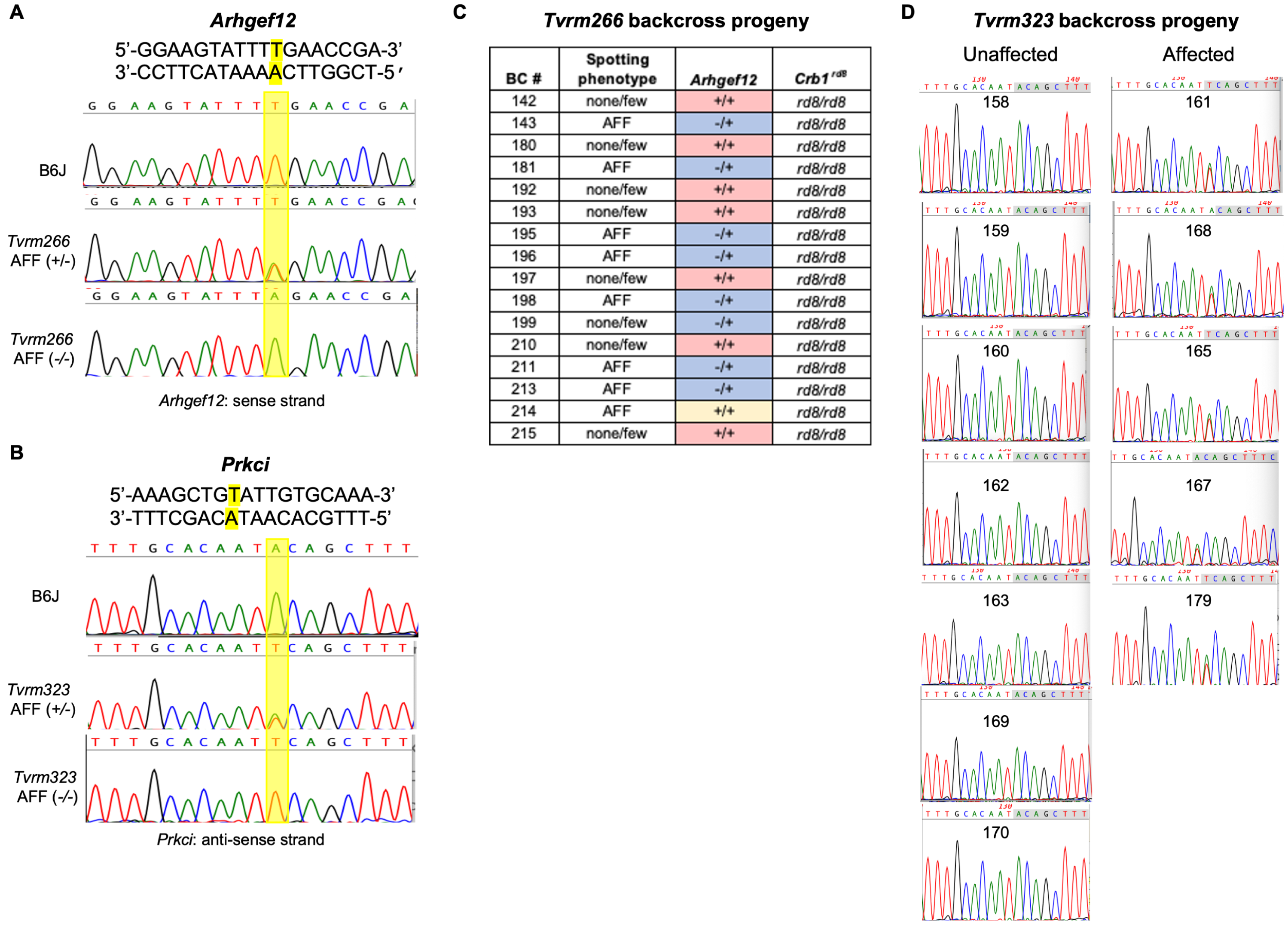

Supplement: S2 Fig — A. Candidate-gene sequencing of the Arhgef12 gene in B6 and affected Tvrm266 mice identified a mutation in both heterozygous and homozygous individuals (yellow highlight). The mutation corresponds to c.71T>A. B. Candidate-gene sequencing of the Prkci gene in B6 and affected Tvrm323 mice identified a mutation in both heterozygous and homozygous individuals (yellow highlight). The sequencing chromatograms shown correspond to the antisense strand. The mutation corresponds to c.406T>A. C. Co-segregation analysis of the disease phenotype with the Arhgef12 mutation in a small mapping cross of Tvrm266 mice with B6N mice. D. Co-segregation analysis of the disease phenotype with the Prkci mutation in the Tvrm323 mapping cross. (TIF) [file pgen.1009798.s002.tif]

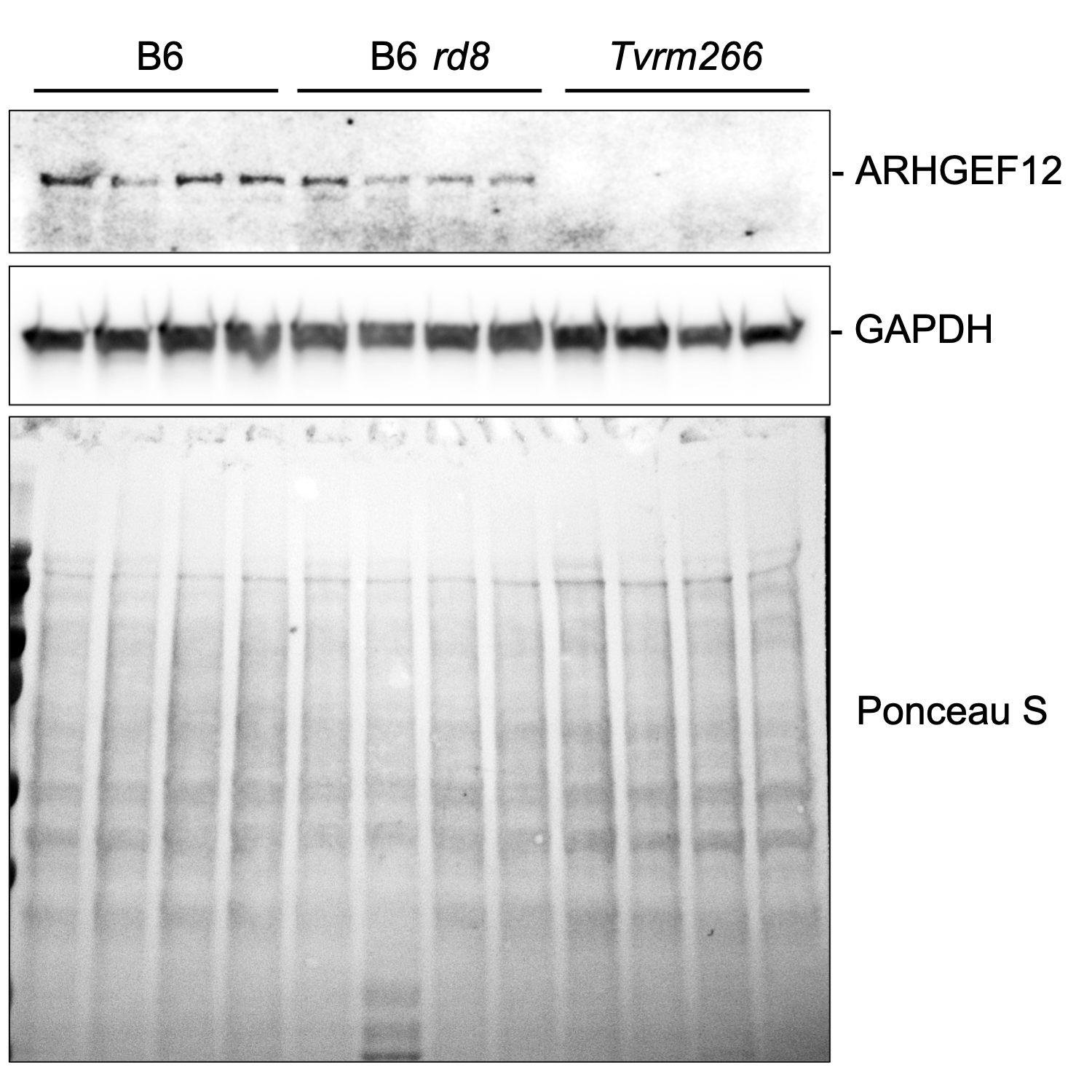

Supplement: S3 Fig — Equal amounts (20 μg) of B6, B6 rd8, and Tvrm266 lysates prepared at one month of age were electrophoresed, transferred to nitrocellulose membranes and probed with antibodies against ARHGEF12 and GAPDH. Ponceau S staining was also performed to demonstrate equivalent loading of total protein. (TIF) [file pgen.1009798.s003.tif]

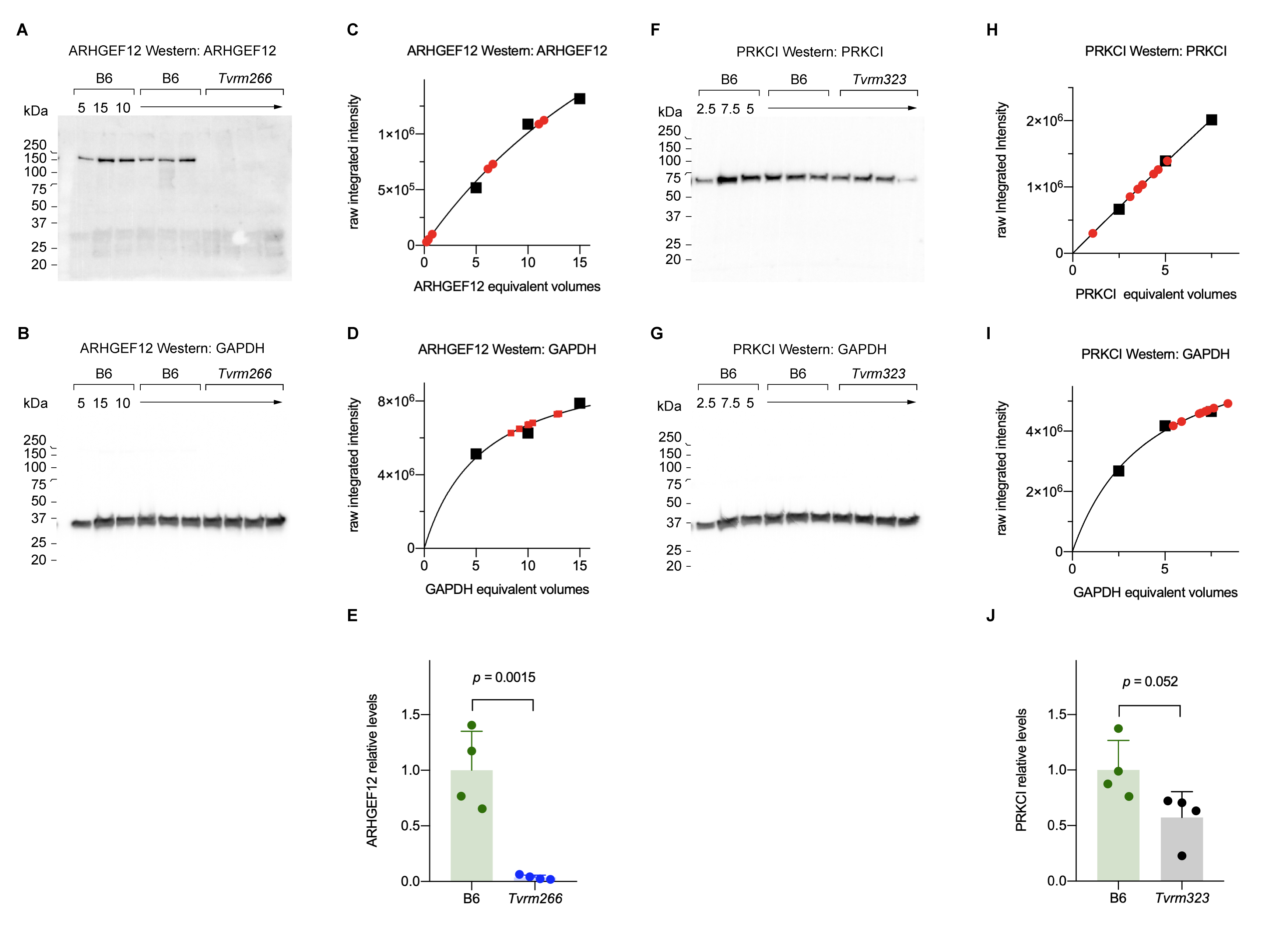

Supplement: S4 Fig — Eyecups were lysed in electrophoresis sample buffer. The volumes loaded for electrophoresis are indicated by the values (in μl) above the blot images. The molecular weights of protein standards are indicated. A–E. ARHGEF12 quantitation. A. ARHGEF12 western blot. B. GAPDH western blot. C. Integrated intensities from lanes shown in panel A. Intensity was measured within a subregion of each lane corresponding to the migration of ARHGEF12 and plotted against sample load volume. Intensities for the B6 standard curve are shown as black squares; the intensities for all 10 μl samples positioned along a hyperbolic fit to the standard curve are shown as red circles. D. Plot of integrated intensity and GAPDH bands from panel B. E. Relative ARHGEF12 levels and statistical analysis. Bars show mean ± SD. F–J. PRKCI quantitation. The blot images and corresponding analyses are identical to those in A–E, except antibody against PRKCI was used in panel F. (TIF) [file pgen.1009798.s004.tif]

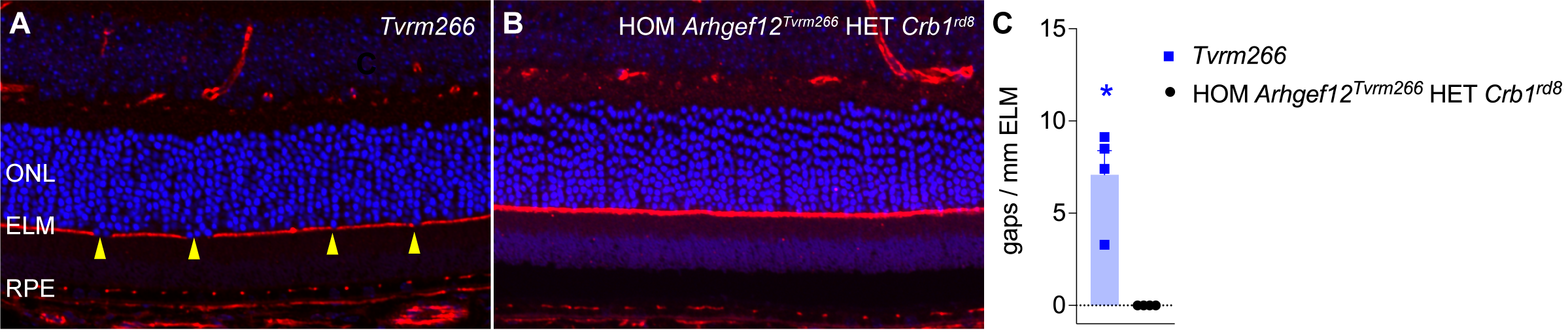

Supplement: S5 Fig — Ocular sections from mice at 10–16 weeks of age (n = 4 for both strains) were stained with DAPI to detect nuclei (blue) and TJP1 to detect the ELM (red). A. Tvrm266 mice at 10 weeks of age. Gaps are indicated with yellow arrows. B. Mice bearing a homozygous Arhgef12Tvrm266 allele and a heterozygous Crb1rd8 allele were obtained from epistasis matings, 10 weeks of age. No gaps were observed. C. Quantitation of gaps per mm of ELM. Bars indicate mean ± SEM. (TIF) [file pgen.1009798.s005.tif]
